# Health Behavior Change after Blood Pressure Feedback

**DOI:** 10.1371/journal.pone.0141217

**Published:** 2015-10-26

**Authors:** Jia Pu, Betty A. Chewning, Heather M. Johnson, David J. Vanness, Henry N. Young, David H. Kreling

**Affiliations:** 1 Palo Alto Medical Foundation Research Institute, Palo Alto, CA, United States of America; 2 University of Wisconsin Madison, School of Pharmacy, Madison, WI, United States of America; 3 University of Wisconsin Madison, School of Medicine and Public Health, Madison, WI, United States of America; 4 University of Georgia, College of Pharmacy, Athens, GA, United States of America; Shanghai Institute of Hypertension, CHINA

## Abstract

Better understanding is needed for antihypertensive medication initiation and lifestyle modification among younger populations with elevated blood pressure. This study aimed to assess health behavior change after receiving a report of elevated blood pressure among African Americans and Caucasians younger than 50 years old. We used the Coronary Artery Risk Development in Young Adults (CARDIA) repository dataset. By examination year twenty, 424 out of 2,478 Caucasian and 2,637 African American participants had received feedback from the CARDIA study due to elevated blood pressure readings. Blood pressure was measured by trained CARDIA researchers at the participant’s home and was repeatedly recorded at seven examinations over twenty years. A feedback/referral letter was sent to participants with an elevated blood pressure reading. On average, participants first had an elevated blood pressure reading at the age of 34. After receiving the feedback letter, 44% of the previously undiagnosed participants received a formal diagnosis. In addition, 23% initiated the use of antihypertensive medication if they had not received medication treatment before. Among the participants with at-risk lifestyle behaviors, 40% reduced alcohol consumption, 14% increased exercise level, 11% stopped smoking, and 8% reached normal weight. While none of the studied patient factors were associated with lifestyle modification, age had a positive impact on antihypertensive medication initiation (p<0.05). We found no evidence of differences in health behavior change between African American and Caucasian participants after receiving the feedback letter. This research is one of the first to study what followed after receiving a feedback letter about elevated blood pressure outside of healthcare settings. Although additional referral care and behavior interventions are needed to facilitate medication initiation and lifestyle modification, our observations suggest that providing blood pressure feedback may have promise as part of a multi-method approach involving blood pressure screening and follow up.

## Introduction

According to the Centers for Disease Control and Prevention, nearly one third (30%) of American adults live with hypertension.[1,2] Despite enormous public health efforts to promote hypertension awareness and management, national studies have found that almost one third (32%) of individuals with hypertension were not aware of their condition, 15% were aware of the condition but were not receiving medication treatment.[3–5] Due to its high prevalence and significance in cardiovascular disease prevention, Healthy People 2020 includes hypertension prevention and control as one of the most critical public health goals of the coming decade.[6]

One important approach to achieve these goals is to initiate guideline-recommended medical treatment in the early stage and address modifiable risk factors early in life to delay or prevent hypertension related complications and comorbidities such as CVD events and chronic renal insufficiency. The current guidelines released by the Joint National Committee on Prevention, Detection, Evaluation, and Treatment of High Blood Pressure (JNC 8) provide specific recommendations to initiate antihypertensive medication and modify lifestyle behaviors in an effort to manage hypertension.[7] However, few studies have targeted elevated blood pressure control in young adults or newly diagnosed populations. There is a need for additional information regarding health behaviors such as antihypertensive medication initiation and lifestyle modification among younger populations with elevated blood pressure. This is particularly true for African Americans considering their elevated risk of hypertension and cardiovascular disease.[8]

Simple screening services such as blood pressure testing and feedback have the potential to motivate lifestyle modification and improve condition awareness and treatment. Thus, this study sought to assess health behavior change after elevated blood pressure was reported to African American and Caucasian individuals younger than 50 years old, with a focus on antihypertensive medication initiation and lifestyle modification. Andersen’s model of health care utilization behavior was adopted in this study to identify factors that could potentially facilitate or impede the health behavior change.[9] The model includes 1) predisposing factors: individual characteristics existing before the health outcomes which suggest the individual’s likelihood to use health services and his/her probability to develop a condition; 2) enabling factors: elements facilitating the access to health care; 3) health needs: the most immediate predictors for the use of health care services and certain health outcomes. Health needs are not included in this study since all the included participants have the health need to control their elevated blood pressure.

**Fig 1:**
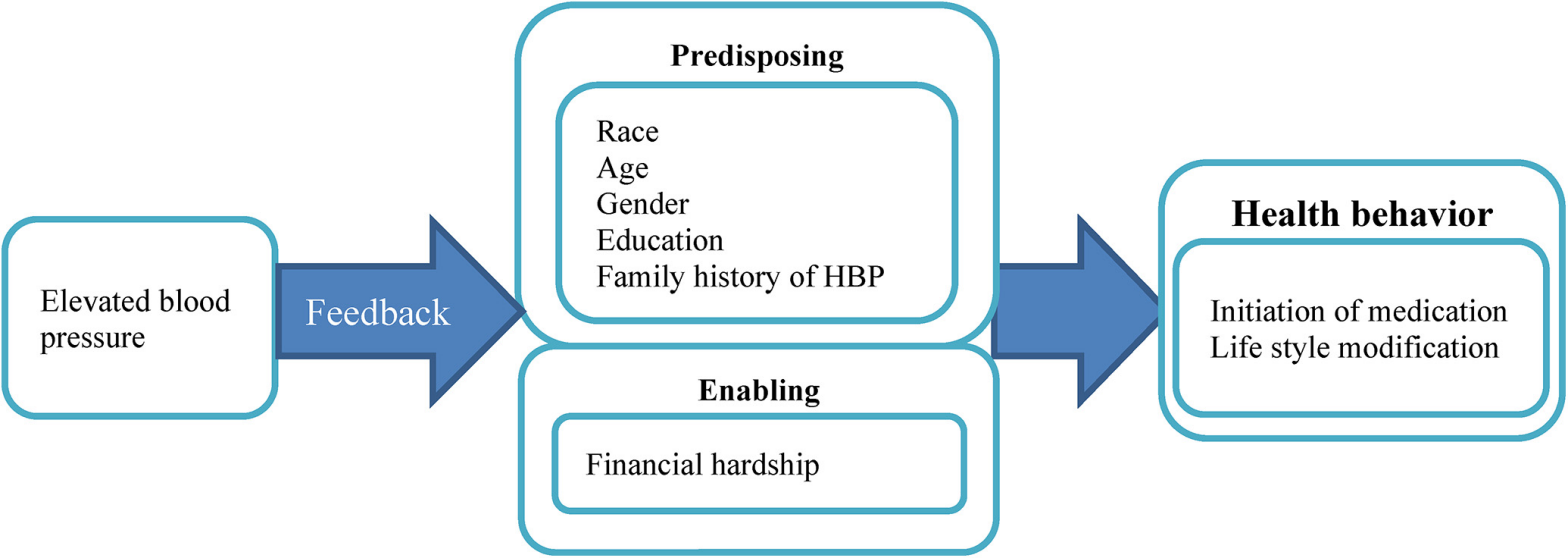
Conceptual model: adopted from Andersen’s model of health care utilization behavior.

As shown by Fig 1, this study proposes participants’ predisposing and enabling factors are associated with health behavior change for blood pressure control, including initiation of antihypertensive medication and lifestyle modification, after receiving feedback on elevated blood pressure.

## Materials and Methods

The study was approved by University of Wisconsin-Madison’s Institutional Review Board. Informed consent was not obtained since all the data were analyzed anonymously.

### Study Cohort

This study used the Coronary Artery Risk Development in Young Adults (CARDIA) repository dataset. The CARDIA study was designed to investigate the natural history of cardiovascular risk in young adults. A total of 5,115 African American and Caucasian Participants were followed up seven times in 1987–1988 (Year 2), 1990–1991 (Year 5), 1992–1993 (Year 7), 1995–1996 (Year 10), 2000–2001 (Year 15), and 2005–2006 (Year 20) which was the latest publicly available wave. Participants ranged from 18 to 30 years old at baseline and were between 38 and 50 years old at year 20; 72% of the total surviving sample remained in the study at year 20.[10]

**Fig 2:**
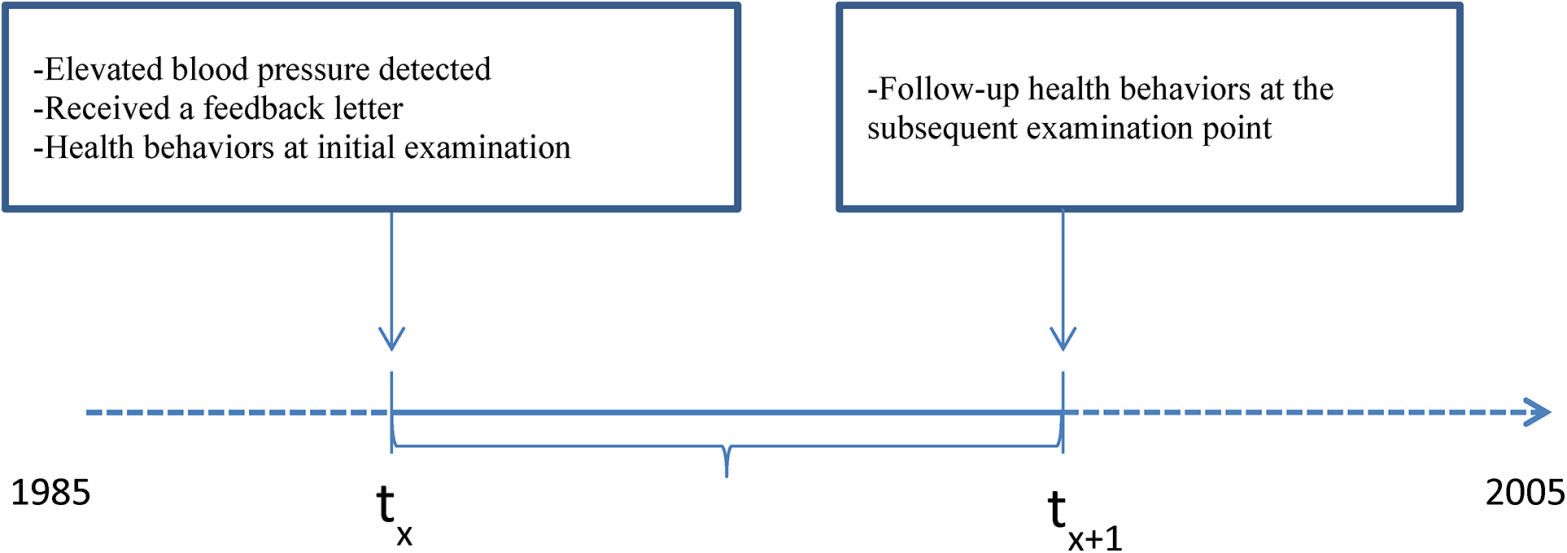
Study design.

As shown in Fig 2, information from two data points in the CARDIA study were used: 1) the initial examination point when an individual’s elevated blood pressure was first recorded; and 2) the subsequent examination point after the initial elevated blood pressure, to explore lifestyle modification and antihypertensive medication use since the receipt of the referral letter. The time interval between the two data points was 2 to 5 years, depending on which wave of the study first observed the participant’s elevated blood pressure. Only participants who participated in both the initial examination and subsequent examination were included in this study. Pregnant participants were excluded from this study.

The CARDIA study measured blood pressure at the participant’s home at each examination point. A unique element of the CARDIA study is that a feedback/referral letter was sent to participants with elevated blood pressure readings (systolic blood pressure ≥140 mm Hg or diastolic blood pressure ≥90 mm Hg), notifying them of this aspect of their health status. The referral letter contained the value of the elevated blood pressure and advised actions, including having a repeat blood pressure evaluation. For those who reported they already had a diagnosis of hypertension, this feedback served as notification of lack of control of their blood pressure. For participants who were not diagnosed before, notification/feedback made them aware of a potential blood pressure problem and alerted them to the need to follow-up with a professional diagnosis and medical care.

### Variables and Analysis

#### Dependent variables: health behavior change

The dependent variables were two main types of health behavior changes: antihypertensive medication initiation and lifestyle modification.

Antihypertensive medication initiation was evaluated by reported antihypertensive medication use at the subsequent examination point after receiving the blood pressure feedback, among those who did not use antihypertensive medication at the initial examination point. A participant was defined as having lifestyle modification (or not) if he/she met one or more of the following criteria:

- Drinking: had at-risk alcohol consumption at the initial examination point based on the National Institute on Alcohol Abuse and Alcoholism drinking categories, but decreased to moderate alcohol consumption or alcohol cessation at the subsequent examination point after the feedback.[11]
- Weight control: was obese or overweight at the initial examination point, but reduced to overweight or normal weight or had more than 5% of weight loss at the subsequent examination point after the feedback; body mass index was measured and calculated by trained CARDIA investigators.
- Smoking: was smoking at the initial examination point, but quit smoking in the subsequent examination point after the feedback.
- Exercise: had low-level exercise at the initial examination point, but increased exercise at the subsequent examination point after the feedback. Exercise level was assessed and calculated using the CARDIA Physical Activity History Questionnaire.[12] Detailed information about CARDIA study design and data collection has been published elsewhere.[10]

#### Independent variables: predisposing and enabling factors

As shown in Fig 1, patient characteristics potentially associated with health behavior change were defined and categorized based on Andersen’s Model. Predisposing factors were measured by participants’ race, age, gender, educational attainment, and family history of hypertension. Enabling factors were assessed by participants’ self-reported financial hardship. All information was collected at baseline and updated at each examination point.

#### Group comparison

We hypothesized that blood pressure feedback could have a different impact on patients who were unaware of their elevated blood pressure previously compared to those who did formerly know they had high blood pressure. Therefore, we classified participants into two groups based on their previous awareness of their blood pressure problem when receiving the feedback on their elevated blood pressure: Group 1 (aware) consisted of participants who reported being diagnosed with hypertension and being informed previously by a health professional, but still had an elevated blood pressure reading in the CARDIA study. Group 2 (not aware) consisted of participants who did not have a hypertension diagnosis and were not aware of elevated blood pressure before, but had an elevated blood pressure reading in the CARDIA blood pressure test.

#### Data analysis

Descriptive analysis and bivariate analyses were used to describe the sample characteristics in the two groups and to understand medication initiation and lifestyle modification in the subsequent years. Multiple logistic regression models were used to explore the associations between the participants’ characteristics, captured by predisposing and enabling factors, and their behavior changes. Statistical significance was set at p<0.05 and 95% confidence intervals were calculated. Statistical analyses were performed using Stata 13 and SAS 9.3.

#### Sensitivity analysis

One important limitation of this study is that elevated blood pressure reading is based on one blood pressure test. According to the National Clinical Guideline Centre, hypertension diagnosis requires repeated examination.[13] Thus, some of the CARDIA participants may have one episode of elevated blood pressure and will not be diagnosed with hypertension. To address this question, we conducted sensitivity analysis within patients with confirmed hypertension using diagnosis information. This included Group 1 participants (reported hypertension diagnosis when elevated blood pressure was first detected) and subpopulation of Group 2 (reported hypertension diagnosis in the subsequent examination after the elevated blood pressure was first detected). Multiple logistic regression models were used to explore the associations between the participants’ characteristics and their lifestyle modification and medication initiation.

## Results

During the 20-year follow-up, 424 participants received feedback from the CARDIA study about elevated blood pressure readings. Among them, 25% were identified at examination year 1, 9% at examination year 2, 10% at examination year 5, 9% at examination year 7, 16% at examination year 10, and 31% at examination year 15.

### Participant characteristics

Participants’ characteristics at the initial examination point when an elevated blood pressure was first recorded are summarized in Table 1. On average, participants first had an elevated blood pressure reading at the age of 34. Two thirds of the studied participants were African Americans. Both African American and Caucasian participants were well educated: 53% of African Americans and 58% of Caucasians received a college education. Most participants had health insurance (82% of African American and 85% of Caucasian participants).

**Table 1:** Participants’ characteristics at baseline (when elevated blood pressure was observed).

	Group 1 Aware	Group 2 Not aware	Overall	P-value
Total number	110	314	424	
**Predisposing**				
Race				0.6470
African Americans	66%	68%	67%	
Caucasians	34%	32%	33%	
Gender				0.0140
Males	50%	63%	60%	
Females	50%	37%	40%	
Age	35	33	34	0.4891
Education				0.0260
High school	30%	37%	35%	
College	60%	53%	55%	
Graduate school	10%	10%	10%	
Family history of hypertension	80%	69%	72%	0.0850
**Enabling**				
Have no financial hardship*	71%	69%	70%	0.7290
**Health behavior variables**				
Use antihypertensive meds	31%	0%	8%	0.0000
Drinking				0.2080
Never drink	21%	19%	19%	
Light drinker	57%	49%	51%	
Moderate drinker	11%	14%	14%	
At-risk drinker	11%	18%	16%	
Weight control/BMI				0.0730
Normal	24%	31%	29%	
Overweight	26%	32%	30%	
Obese	50%	37%	41%	
Smoking				0.7060
Never smoked	61%	61%	61%	
Former smoker	15%	12%	13%	
Current smoker	25%	27%	26%	
Exercise				0.1180
Low	49%	38%	40%	
Low to moderate	25%	25%	25%	
Moderate to high	13%	21%	19%	
High	13%	16%	15%	

Have no financial hardship was defined by the answer of “not very hard” to the question of “How hard is it for you (and your family) to pay for the very basics like food and heating?” P-values were based on pair-wise chi-square test for categorical variables and pairwise t test for continuous variables.

Among the participants selected for study, 110 reported being diagnosed with hypertension before but still had elevated readings in the CARDIA blood pressure test/Group 1 (aware), and 314 participants were not aware of their potential blood pressure problem before the elevated readings in the CARDIA blood pressure test/Group 2 (not aware).

Comparison between the two groups revealed a significant difference in participants’ gender and educational attainment. Participants in the previously undiagnosed Group 2 were more likely to be male (63% versus 50% in Group 1, p<0.05) and less likely to have a college degree (53% versus 60% in Group 1, p<0.05). More than two-thirds (approximately 70%) of both groups reported having no financial hardship. There was no significant difference of race or financial hardship between the two groups.

In terms of participants’ health behaviors, 31% of the participants in Group 1 were using antihypertensive medication in the initial examination. As expected, due to unawareness of their elevated blood pressure, none of the participants in Group 2 were taking antihypertensive medication. A total of 16% of the studied participants reported at-risk alcohol consumption, 71% were overweight or obese, 26% were current smokers, and 40% reported low-level exercise in the initial examination.

### Health behavior change in the subsequent examination

Table 2 shows antihypertensive medication initiation and lifestyle modification among participants with one or more risk behaviors at the initial examination point, in both Group 1 and Group 2. Twenty three percent of those who did not use antihypertensive medication initiated drug therapy after receiving the feedback letter. In addition, 44% of the previously undiagnosed participants gained a formal diagnosis of hypertension. The biggest lifestyle behavior change was alcohol consumption; overall, 40% of at-risk drinkers reported lower, not at risk levels of drinking after receiving the feedback letter. This change was more than double the impact on the percent of individuals with a change in lifestyle from feedback for the other lifestyle behaviors: 18% of participants with overweight or obesity improved their body weight after receiving the feedback letter, 11% of smokers stopped smoking, and 14% of participant with low level of exercise reached normal or high level of exercise. The systolic blood pressure and the diastolic blood pressure at the subsequent examination point after receiving the feedback letter was 129/86 mm Hg for Group 1 and 126/82 mm Hg for Group 2 respectively. It shows 44% of participants in Group 1 and 29% in Group 2 had elevated blood pressure at the subsequent examination point, putting them potentially at elevated risk for hypertension related complications.

**Table 2:** Health behavior changes after the feedback by comparison groups.

	Group 1	Group 2	Overall
	Aware	Not aware	
Antihypertensive meds (N of did not use)	76	314	390
Initiated, No. (%)	25 (33%)	63 (20%)	88 (23%)
Drinking (N of at-risk drinkers)	12	56	68
Modified, No. (%)	4 (33%)	23 (41%)	27 (40%)
Weight control (N of overweight or obese)	84	217	301
Modified, No. (%)	16 (19%)	37 (17%)	53 (18%)
Smoking (N of current smokers)	27	85	112
Modified, No. (%)	4 (15%)	8 (9%)	12 (11%)
Exercise (N of light level exercise)	53	117	170
Modified, No. (%)	5 (9%)	18 (15%)	23 (14%)
Diagnosis (N of no previous diagnosis)	0	314	314
Diagnosed, No. (%)		138 (44%)	138 (44%)
Average blood pressure readings at the subsequent examination			
Systolic blood pressure, mm Hg (95% CI)	129 (126, 132)	126 (124, 128)	127 (125, 128)
Diastolic blood pressure, mm Hg (95% CI)	86 (83, 88)	82 (81, 83)	83 (82, 84)
Patients with elevated blood pressure at the subsequent examination			
Proportion, % (95% CI)	44 (35, 54)	29 (25, 34)	33 (29, 38)

Ns are the numbers of study participants in the initial observation with the at risk behavior.

### Participants’ characteristics and health behavior change

Based on the multiple logistic regression results (Table 3), the likelihood of adopting antihypertensive medication significantly increased with age (p<0.01). Additional analyses were conducted to explore potential factors contributing to the observed association between age and medication adoption. Comparison was made between those who were younger than 35 years old (N = 224) to those aged 35–50 (N = 166). A total of 10% of those who were younger than 35 years adopted antihypertensive medication while the medication initiation rate was 40% among the 35–50 years. There were no significant differences of race, family history of hypertension, educational attainment or financial hardship between the two age groups based on multiple logistic regression. However, the younger age group was significantly more likely to be male.

**Table 3:** Odds ratio of behavior changes by participants’ characteristics in the subsequent examination.

Modified vs. Not	Medication	Lifestyle
**Total**	390	375
Modified, No. (%)	88 (23%)	106 (28%)
**Predisposing**		
Race		
African Americans	Ref	Ref
Caucasians	0.76	1.03
Gender		
Males	Ref	Ref
Females	1.31	0.98
Age	1.16**	1.02
Education		
High school	Ref	Ref
College	1.02	0.76
Graduate school	0.68	0.54
Family history of hypertension		
No	Ref	Ref
Yes	1.76*	1.01
**Enabling**		
Have financial hardship		
Yes	Ref	Ref
No	0.80	0.60

*statistically significant p<0.1

**p<0.05 compared with reference group, by multiple logistic regression.

Multiple logistic regression models were used to explore participants’ characteristics associated with the lifestyle modification (Table 3). A total of 375 participants (88%) had one or more risk behaviors in the initial examination and 106 participants (28%) changed at least one risk behavior as reported at the subsequent study examination (with 15 of these participants changing more than one risk behavior). None of the studied characteristics showed a statistically significant likelihood of modifying lifestyle.

Sensitivity analysis was conducted among patients with diagnosed blood pressure (Table 4), including all participants in Group 1 (n = 110) and 136 participants in Group 2 (44%). Consistent with the primary analysis results, none of the studied participants’ characteristics was associated with lifestyle modification. Beyond age, initiation of antihypertensive medication was also associated with African American race among participants with diagnosed hypertension.

**Table 4:** Sensitivity analysis: odds ratio of behavior changes by participants’ characteristics in the subsequent examination (diagnosed patients only).

Modified vs. Not	Medication	Lifestyle
**Total**		
Modified, No. (%)	88 (42%)	64 (29%)
**Predisposing**		
Race		
African Americans	Ref	Ref
Caucasians	0.37*	0.89
Gender		
Males	Ref	Ref
Females	0.86	0.84
Age	1.25**	1.02
Education		
High school	Ref	Ref
College	0.86	0.57
Graduate school	0.37	0.54
Family history of hypertension		
No	Ref	Ref
Yes	1.76	1.73
**Enabling**		
Have financial hardship		
Yes	Ref	Ref
No	0.88	0.64

*statistically significant p<0.1

**p<0.05 compared with reference group, by multiple logistic regression.

## Discussion

This research is one of the first to study what followed after receiving a feedback letter about elevated blood pressure from the CARDIA study. Further it does so with a sample carefully constructed to allow comparisons of Caucasian and African American populations of a fairly comparable socioeconomic status. Most of the previous studies on hypertension awareness and blood pressure management were conducted at health care settings or were based on cross-sectional surveys such as NHANES, which did not follow behavior change in response to blood pressure evaluation. This study allowed for a careful longitudinal evaluation of health behavior change as an effort to control elevated blood pressure using a comparable biracial cohort.

We found receiving feedback about elevated blood pressure was associated with an increase in the use of antihypertensive medication and receiving a formal diagnosis of hypertension for both African Americans and Caucasian participants. There was also a modest reduction in the lifestyle risk factors in the subsequent examination. Among the predisposing and enabling factors, age was the only variable significantly associated with the initiation of antihypertensive medication at the subsequent examination point. This is consistent with results in previous studies using data from a large health care system.[14,15] Young adults were found to have delayed diagnosis of hypertension and medication initiation even with regular primary care. Together our findings with existing literature suggest tailored interventions be provided to young adults to address barriers in hypertension diagnosis and treatment and to physicians to initiate therapy in hypertensive young adults. Although number needed to prevent a CVD event is higher in young adults compared to older people, it is important to provide early treatment in order to prevent or to delay hypertension related complications and comorbidities.

### Differences between Group 1 and Group 2

Our study found a significant difference in gender and educational attainment between those who were aware of their hypertension (Group 1) and those who were not aware of their hypertension when elevated blood pressure was reported (Group 2). Those in the unaware group were more likely to be male and had lower educational attainment, which is consistent with previous studies. Using the NHANES III data, Hyman et al reported that males tended to have lower awareness of their hypertension and untreated hypertension.[4] Our study reinforces this gender difference in hypertension awareness and management in a younger population. Gender differences in health beliefs, contact with health care providers and other health factors may contribute to this observation. Further research is needed to better understand this gender difference in order to provide gender-specific interventions in hypertension awareness and management.

Besides gender differences, previous study also found a racial disparity in hypertension awareness and blood pressure control.[16] To our knowledge, no existing literature has examined potential racial differences in health behavior change after blood pressure feedback. In this study, we did not find any significant differences in the likelihood to change health behaviors between African Americans and Caucasians after receiving a report on their elevated blood pressure. Both groups showed significant progress in health care seeking behaviors including medication treatment, and modest change with lifestyle modification. The absence of health disparities in this study can be potentially explained by socioeconomic status and education level. Both African American and Caucasian participants were well educated-all participants received high school or higher education, and both groups had a high percentage of insured participants.

### Health behavior change in the subsequent examination

This study assessed behavior change by the subsequent study examination point, which was 2–5 years after receiving the report on elevated blood pressure. We found an increase in the use of antihypertensive medication in both African American and Caucasian participants. This suggests the feedback letter may promote health care seeking behaviors. According to the Health Belief Model, *cues to action* or strategies to activate readiness could trigger action. In this case, a simple referral letter may have provided a “cue to action” by increasing participants’ awareness of their medical condition and motivating them to seek medical care.[17] This finding is particularly important for African Americans considering their elevated risk for hypertension and cardiovascular diseases. Receiving a timely diagnosis and initiating antihypertensive medication at an early stage could limit the progression of elevated blood pressure and potentially reduce the burden of CVD among African Americans.

The largest change in at-risk behaviors in this study was that 40% of the at-risk drinkers reduced their alcohol consumption after the baseline examination letter identifying their elevated blood pressure. The alcohol consumption finding is particularly interesting in that it was not tested in earlier studies (Kip et al, 2002; Johnson et al, 2011).[18,19] There is a need to replicate the alcohol risk behavior finding in further research.

The finding of limited change in smoking, weight control and exercise is consistent with previous studies. A prior study of CARDIA participants found no change in smoking, weight control or physical activity among both African American and Caucasian participants after the occurrence of a heart attack or stroke in an immediate family member.[18] A study on long-term effects of carotid screening also found a lack of lifestyle change among Caucasian participants after receiving intensive counseling on carotid ultrasound screening results and recommendations.[19] These findings indicate that one-time feedback or education is not enough to promote many long-term lifestyle modifications, but may have bigger impact on alcohol consumption. Future studies are needed to examine the potential for prolonged, multipronged and tailored approaches to influence lifestyle modification among younger populations with elevated blood pressure.

Among the studied patients’ characteristics, age was significantly associated with the initiation of antihypertensive medication, while none of other factors was associated with lifestyle modification. Although the cause for this association is unknown, we found males were significantly more likely to have elevated blood pressure at a younger age (<35 years). It is possible that younger males are less likely to have doctor visits and/or to have more difficulty adopting antihypertensive medication. Further studies should be conducted in this age and gender group to better understand their needs and barriers in hypertension diagnosis and management. Gender and age differences in the onset of hypertension also need to be further examined. In addition, among participants with diagnosed hypertension, we found that African Americans were more likely to start antihypertensive medication. This is consistent with findings from the Multi-Ethnic Study of Atherosclerosis (MESA), which may be a reflection of health care provider’s awareness of higher burden of hypertension among African Americans resulting in prescription writing and decisions by African American patients to follow through with prescription acquisition, dosing, and adherence.[20]

### Limitations

Several study limitations should be noted. This is an observational study. So no causal relationship can be established. This study had a relatively small sample size, which limits the study design, the analysis methods and the power to detect significant differences. Several measures, including diagnosis of hypertension, were self-reported and are subject to self-report bias. However, previous work on reasons for hospitalization indicated good concordance between self-reported hospitalization history and the medical records among CARDIA participants.[21] In addition, elevated blood pressure readings were based on blood pressure test results at each data point. Thus, some of the CARDIA participants may only have one episode of elevated blood pressure and will not be diagnosed with hypertension. However, sensitivity analysis with diagnosed participants found consistent results. In addition, among the participants who had elevated blood pressure in the CARDIA blood pressure test, 44% of them reported being diagnosed by health professionals in the subsequent examination.

### Conclusion

This study suggests that a simple blood pressure feedback letter has the potential to improve condition awareness and blood pressure management in a younger population, in particular the initiation of antihypertensive medications. This appears equally true across racial and gender groups. Building on this finding, future programs could explore feedback in many forms such as providing people with their blood pressure readings at a clinic visit, in a medical electronic message or letter, at a pharmacy, or through other means.
